# Prognostic Value of EEF1A1 and Its Correlation with Immune Regulation in Kidney Renal Clear Cell Carcinoma

**DOI:** 10.3390/cancers18182938

**Published:** 2026-09-10

**Authors:** Qiang Yuan, Xinmiao Ma, Sensen Ruan, Yu Zhang, Xiancheng Li

**Affiliations:** Department of Urology, The Second Affiliated Hospital of Dalian Medical University, Dalian 116023, China; q36073906@gmail.com (Q.Y.); maxinmiao1003@163.com (X.M.); rss19890592730@163.com (S.R.); zy13464668079@163.com (Y.Z.)

**Keywords:** EEF1A1, kidney renal clear cell carcinoma, prognosis, survival, immune infiltration

## Abstract

Background: Eukaryotic translation elongation factor 1 alpha 1 (EEF1A1) primarily participates in protein synthesis by binding aminoacyl-tRNA complexes to facilitate peptide chain elongation on ribosomes. Its expression and functional roles exhibit significant heterogeneity across various malignancies, exerting dual regulatory effects as both an oncogene and a tumor suppressor. This study aims to investigate the potential prognostic value and tumor-suppressive role of EEF1A1 in kidney renal clear cell carcinoma (KIRC). Methods: We analyzed the differential expression of EEF1A1 in KIRC and its correlation with patient prognosis based on the TCGA, GEO, and HPA databases. The STRING and GEPIA databases were utilized to perform functional enrichment analysis of its interacting proteins and co-expressed genes. The xCell algorithm was employed to assess the correlation between EEF1A1 and immune cell infiltration, immune checkpoints, and immunomodulatory molecules. Furthermore, drug sensitivity analysis was conducted to evaluate its clinical application potential. Finally, the expression of EEF1A1 in 786-0 and A498 cell lines was validated via qRT-PCR and Western blotting. Furthermore, CCK-8, wound healing, and Transwell migration/invasion assays were performed to evaluate cell proliferation, migration, and invasion, respectively. Results: EEF1A1 may function as a negative regulator of malignant behaviors in KIRC tissues and cell lines, and its expression level was closely associated with clinicopathological features and prognosis of patients. GO, KEGG, and GSEA enrichment analyses revealed that low EEF1A1 expression is closely linked to immunosuppressive pathways. Further immunological analysis confirmed significant correlations between EEF1A1 and various immune cell infiltrates, immune checkpoints, tumor-infiltrating lymphocytes, and immunomodulatory molecules. Moreover, cells with high EEF1A1 expression exhibited increased sensitivity to anti-tumor drugs, with expression levels negatively correlated with inhibitory activity (IC50). Finally, overexpression of EEF1A1 significantly inhibited the proliferation, migration, and invasion of clear cell renal cell carcinoma cells. Conclusions: EEF1A1 serves as a potential prognostic biomarker in KIRC and is associated with clinical progression, immune-related characteristics, metabolic pathways, and drug sensitivity. Functional validation further supports its role in regulating malignant phenotypes of KIRC cells.

## Simple Summary

In this study, we comprehensively analyzed transcriptomic data from patients with kidney renal clear cell carcinoma (KIRC) obtained from the TCGA and GEO databases. We identified that EEF1A1 exhibits cohort-dependent expression patterns in KIRC, with differential expression profiles observed among independent datasets. Furthermore, EEF1A1 expression was significantly associated with advanced TNM stage, higher pathological grade, and unfavorable clinical outcomes in KIRC patients. Functional enrichment analyses revealed that EEF1A1-interacting and co-expressed genes are primarily involved in multiple metabolic and immune regulatory pathways. Moreover, EEF1A1 expression was also significantly correlated with the infiltration levels of various immune cell populations within the KIRC tumor microenvironment. In addition, differential drug sensitivity analyses indicated that EEF1A1 expression is significantly associated with the sensitivity of KIRC cells to several commonly used chemotherapeutic and targeted agents, including sorafenib, paclitaxel, and cisplatin. Finally, our in vitro experiments validated the expression pattern of EEF1A1 in clear cell renal cell carcinoma and further confirmed its functional role in regulating the biological behaviors of renal cancer cells.

## 1. Introduction

Kidney renal clear cell carcinoma (KIRC), an adenocarcinoma originating from the tubular epithelial cells of the renal parenchyma, is among the most prevalent malignant tumors worldwide. It accounts for approximately 70–80% of all renal cell carcinoma (RCC) cases and represents the most clinically aggressive histological subtype [1]. Despite significant advancements in diagnostic and therapeutic modalities in recent years, the incidence and mortality rates of KIRC remain high, with a mortality rate ranging from 30% to 40% [2]. Consequently, identifying effective early-stage prognostic biomarkers and discovering novel therapeutic targets are of paramount clinical importance for improving early diagnosis and patient outcomes in KIRC. Eukaryotic translation elongation factor 1 alpha 1 (EEF1A1) is the second most abundant protein in cells after actin and serves as a core subunit of the eukaryotic elongation factor 1 (EEF1) complex. Its canonical translation function involves binding with GTP to form a complex that facilitates the delivery of aminoacyl-tRNAs to the ribosome, thereby promoting polypeptide chain elongation [3]. Beyond its classical role in translation, EEF1A1 is extensively involved in critical biological processes, including cytoskeleton remodeling, DNA damage repair, signal transduction, and tumor immune evasion. It plays a pivotal regulatory role in the proliferation, migration, invasion, and drug resistance of various cancer cells [4,5,6,7,8,9,10]. Previous studies have demonstrated the diverse oncogenic roles of EEF1A1 across multiple malignancies. In pancreatic cancer, EEF1A1 interacts with FBXO32 to activate FAK signaling, thereby driving cancer progression and metastasis [11]. In hepatocellular carcinoma, it activates the transcription factor SP1, which upregulates HGF and triggers the PI3K/AKT pathway to promote epithelial–mesenchymal transition (EMT) and metastasis [12]. In non-small-cell lung cancer (NSCLC), EEF1A1 binds to the long non-coding RNA CRYBG3, facilitating its nuclear translocation and the subsequent upregulation of MDM2; this enhances cell migration via the MDM2/MTBP/ACTN4 axis [13]. Conversely, in triple-negative breast cancer (TNBC), the binding of EEF1A1 with penicillide-like compounds inhibits the expression of downstream effectors RPL27A and RPLP0, leading to the induction of apoptosis and the suppression of invasion and migration [14].

However, the specific role of EEF1A1 in KIRC remains poorly understood. In this study, we performed a systematic bioinformatic analysis to investigate the prognostic value, clinical features, biological functions, and immune infiltration patterns of EEF1A1 in KIRC, as well as its correlation with drug sensitivity. Subsequently, the expression levels of EEF1A1 were validated in 786-0 and A498 cell lines using qRT-PCR and Western blotting. Finally, we established 786-0 and A498 cell lines with stable EEF1A1 overexpression. CCK-8, wound healing, and Transwell migration and invasion assays were performed to evaluate the effects of EEF1A1 on the proliferation, migration, and invasion of KIRC cells.

## 2. Materials and Methods

### 2.1. Data Collection and Preprocessing

Transcriptomic profiles and corresponding clinical data for 601 KIRC samples (529 tumor tissues vs. 72 normal tissues) were retrieved from the TCGA database (https://cancergenome.nih.gov/ (accessed on 25 April 2025)). For external validation, three independent datasets (GSE66272, GSE36895, and GSE53757) were downloaded from the GEO database (https://www.ncbi.nlm.nih.gov/geo/ (accessed on 17 May 2025)).

### 2.2. Pan-Cancer Expression Analysis of EEF1A1

The mRNA expression levels of EEF1A1 across various cancer types in the TCGA pan-cancer cohort were evaluated using the Gene_DE module of TIMER2.0 (http://timer.cistrome.org/ (accessed on 17 May 2025)). Differential expression between tumor and corresponding normal tissues was further corroborated using the Xiantao Academic Platform.

### 2.3. Expression and Clinicopathological Correlation Analysis in KIRC

The expression differences in EEF1A1 between KIRC and normal tissues were analyzed and visualized using R4.5.2 packages (ggplot2 v4.0.1; ggpubr v0.6.2). The correlation between EEF1A1 expression and clinicopathological parameters—including age, gender, TNM stage, and pathological grade—was investigated. Protein expression levels and subcellular localization were evaluated via immunohistochemistry (IHC) staining data from the Human Protein Atlas (HPA) (https://www.proteinatlas.org/ (accessed on 18 May 2025)).

### 2.4. Prognostic Value and DNA Methylation Analysis

Survival analysis was performed using the survival (v3.8.6) and rms (v8.1.0) R packages. The impact of EEF1A1 expression on overall survival (OS), disease-specific survival (DSS), and progress-free interval (PFI) was assessed using Kaplan–Meier curves and univariate/multivariate Cox regression analyses. A nomogram model was constructed to predict survival probability, with accuracy validated by calibration curves. Furthermore, the relationship between EEF1A1 methylation levels and prognosis was explored using the MethSurv database.

### 2.5. PPI Network, Hub Gene Identification, and Functional Enrichment

Protein–protein interaction (PPI) partners were predicted using the STRING database (https://www.string-db.org/ (accessed on 21 May 2025)) with a confidence score threshold of 0.40 and a maximum of 40 interactors. The network was visualized using Cytoscape3.7.1 software. The top 100 co-expressed genes were identified via the GEPIA database (http://gepia.cancer-pku.cn/), and the CytoHubba plugin was employed to extract core (Hub) genes. Correlation analysis between EEF1A1 and these Hub genes was conducted. Functional characterization was performed through GO and KEGG enrichment analyses of co-expressed genes, supplemented by Gene Set Enrichment Analysis (GSEA) based on TCGA-KIRC data to identify associated biological processes and signaling pathways.

### 2.6. Immune Infiltration, Checkpoints, and Immunomodulatory Analysis

The xCell algorithm was utilized to estimate the correlation between EEF1A1 expression and the infiltration levels of 33 immune cell types. The ESTIMATE algorithm was applied to calculate Immune, Stromal, and ESTIMATE scores. Relationships between EEF1A1 and immune checkpoints, tumor-infiltrating lymphocytes, immunomodulators (inhibitors, stimulators, and MHC molecules), and chemokines/receptors were analyzed using R and the TISIDB database.

### 2.7. Drug Sensitivity Analysis

Given that EEF1A1 is involved in reshaping the tumor immune microenvironment and immune status is a critical modulator of therapeutic response in ccRCC, we further explored the association between EEF1A1 expression and pharmacological sensitivity. Pharmacological sensitivity data were retrieved from the CellMiner™ database. After normalization, the Wilcoxon rank-sum test was applied to compare the half-maximal inhibitory concentration (IC50) of GDSC-derived drugs between the high- and low-EEF1A1 subgroups. Spearman correlation analysis was conducted to screen compounds whose inhibitory potency exhibited significant correlations with EEF1A1 expression levels.

### 2.8. Cell Culture and Establishment of Stable EEF1A1-Overexpressing Cell Lines

786-0 and A498 cells were cultured in RPMI-1640 and MEM media, respectively, both supplemented with 10% fetal bovine serum (FBS). The stable EEF1A1-overexpressing KIRC cell lines and their corresponding negative controls were constructed by Xiamen Yimo Biotech Co., Ltd. (Xiamen, China). Briefly, the full-length human EEF1A1 coding sequence was cloned into the pcDNA3.1 eukaryotic expression vector. The recombinant plasmids and empty vectors were then stably transfected into 786-0 and A498 cells, followed by selection with puromycin to obtain stable cell clones. The efficiency of EEF1A1 overexpression was validated via qRT-PCR and Western blotting. The primer sequences used are listed in Table 1. The human renal cancer cell lines 786-0 and A498, as well as the human renal proximal tubular epithelial cell line HK-2, were obtained from the American Type Culture Collection (ATCC, Manassas, VA, USA) and maintained according to the recommended protocols. All cell cultures were routinely tested for Mycoplasma contamination by PCR-based methods before experimental procedures, and no contamination was detected.

### 2.9. qRT-PCR and Western Blotting

qRT-PCR and Western blot were performed to detect the mRNA and protein expression levels of EEF1A1 in KIRC cell lines (786-0 and A498) and normal renal tubular epithelial cells (HK-2), so as to verify the results of bioinformatics analysis. After constructing EEF1A1-stably overexpressing cell lines (786-0 and A498), the overexpression efficiency was evaluated using the same methods.

Total RNA was extracted from the above cells and reverse-transcribed into first-strand cDNA for subsequent qRT-PCR analysis. Amplification was performed using a QuantStudio 5 (Q5) real-time PCR system under the following conditions: pre-denaturation at 95 °C for 120 s, followed by 40 cycles of denaturation at 95 °C for 15 s, annealing at 60 °C for 15–30 s, and extension at 72 °C for 30 s. The relative gene expression level was calculated using the 2^−ΔΔCT^ method.

Western blotting was performed after qRT-PCR. Cells (786-0, A498, and corresponding EEF1A1-overexpressing cells) were collected and lysed using lysis buffer to extract total protein. Protein samples were mixed with loading buffer, denatured by boiling, separated by SDS-PAGE, and then transferred onto 0.45 µm PVDF membranes. The membranes were blocked with 5% bovine serum albumin (BSA) to reduce non-specific binding, followed by incubation with EEF1A1-specific primary antibodies. After washing, the membranes were incubated with horseradish peroxidase (HRP)-conjugated secondary antibodies. Finally, protein signals were detected by film exposure using enhanced chemiluminescence (ECL) reagent.

### 2.10. Cell Viability Assay (CCK8)

EEF1A1-stably overexpressing 786-0 and A498 cells were seeded into 96-well plates at a density of 2000 cells per well, with triplicate wells set for each group. At 0, 24, 48, 72, and 96 h post-seeding, 10 µL of CCK-8 reagent was added to each well, followed by incubation for 2 h. The absorbance (OD value) at 450 nm was measured using a microplate spectrophotometer (SpectraMax® 190, Molecular Devices, San Jose, CA, USA). Cell growth curves were subsequently constructed, and cell viability in each group was calculated accordingly.

### 2.11. Wound Healing Assay

EEF1A1-stably overexpressing 786-0 and A498 cells were seeded into 6-well plates and cultured until they reached approximately 90% confluence. Three to four parallel scratches were made across each well using a 200 µL pipette tip. After washing away detached cells with sterile PBS, the medium was replaced with fresh serum-free medium. Images of the scratched areas were captured using an inverted microscope (Leica DMi1, Leica Microsystems, Wetzlar, Germany) at 0 and 24 h after scratching.

### 2.12. Transwell Migration and Invasion Assay

EEF1A1-stably overexpressing 786-0 and A498 cells were subjected to serum starvation for 12 h, then trypsinized and resuspended in serum-free medium to a density of 1 × 10^5^ cells/mL. For the migration assay, 200 µL of cell suspension was added to the upper chamber, while the lower chamber was filled with 600 µL of complete medium containing 10% FBS. For the invasion assay, the bottom of the insert was pre-coated with 70 µL of diluted Matrigel and incubated at 37 °C for 1 h to solidify before the addition of the cell suspension. Both assays were incubated at 37 °C for 24 to 48 h. After incubation, the inserts were washed with PBS, fixed with 4% fixative for 20 min, and stained with 0.1% crystal violet for 20 min. After drying, images were captured and saved using an inverted microscope.

### 2.13. Statistical Analysis

The *t*-test was used to analyze the expression of EEF1A1 in KIRC tissues. Significant differences between three or more groups were analyzed by one-way analysis of variance (ANOVA). The area under the ROC curve was calculated to assess the diagnostic value of EEF1A1 for KIRC. Cox regression, Kaplan–Meier survival analysis and a log-rank test were used to analyze the prognostic value of EEF1A1. Pearson correlation analysis was applied to explore the relationship between EEF1A1 expression and KIRC immune cell infiltration. The figures were graphed using GraphPad Prism 9.5 and R language. A statistical significance level of *p* < 0.05 was considered statistically significant.

## 3. Results

### 3.1. Expression Characteristics of EEF1A1 in Pan-Cancer and KIRC

**Figure 1:**
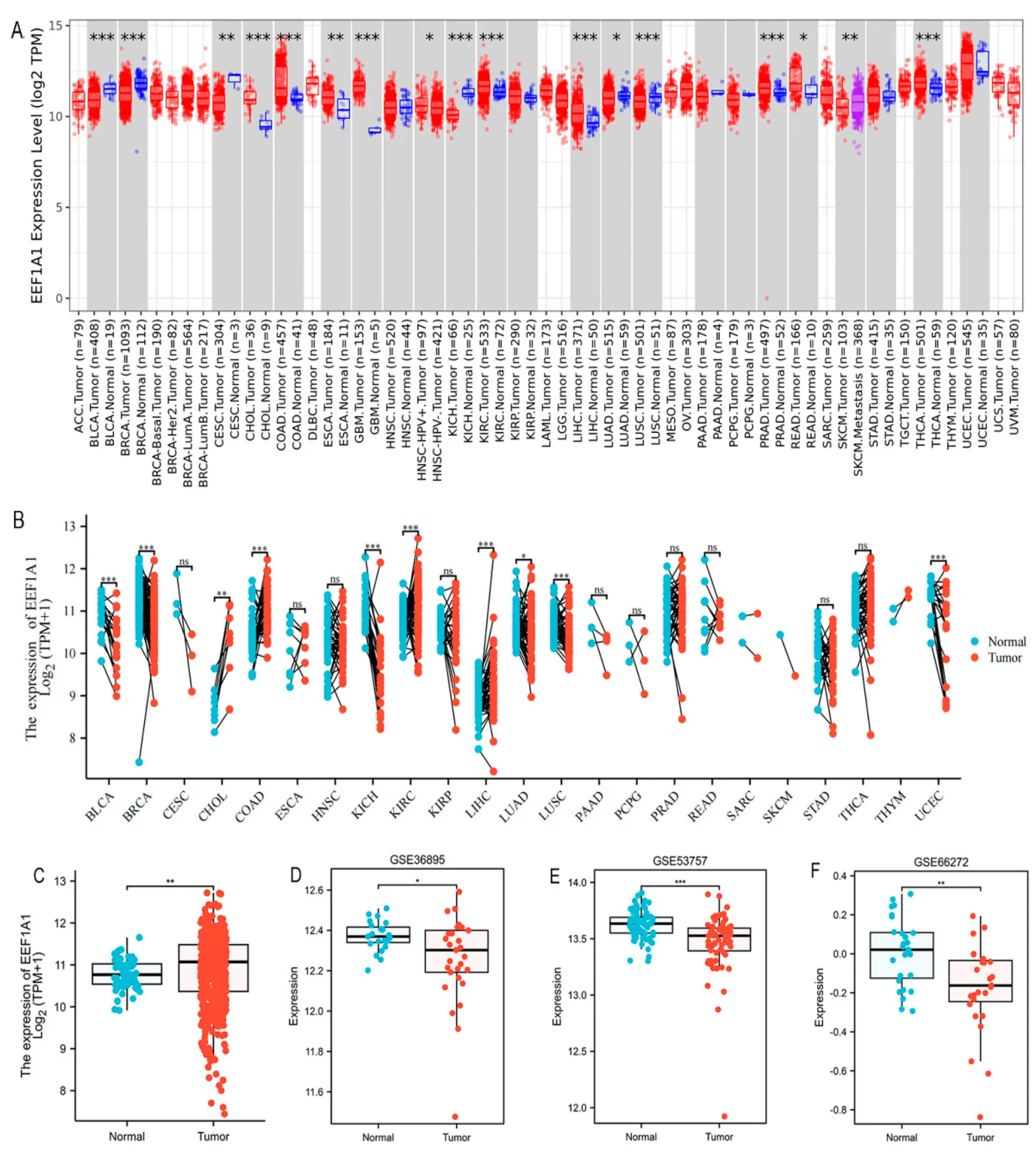
Expression of EEF1A1 in pan-cancer. (**A**) Expression levels of EEF1A1 in various types of cancers based on the TIMER2.0 database; (**B**) comparative analysis of EEF1A1 expression in 23 tumor tissues and normal paired normal tissues; (**C**) expression differences in EEF1A1 in KIRC and paracancerous tissues in the TCGA database; (**D**–**F**) expression differences in EEF1A1 in KIRC and paracancerous tissues in three different datasets based on the GEO database. * *p* < 0.05, ** *p* < 0.01, *** *p* < 0.001, ns, no significant difference.

Pan-cancer analysis revealed that EEF1A1 was significantly overexpressed in 10 tumor types, including rectum adenocarcinoma (READ), cholangiocarcinoma (CHOL), colon adenocarcinoma (COAD), and liver hepatocellular carcinoma (LIHC), while significantly downregulated in 7 others, including bladder urothelial carcinoma (BLCA), lung adenocarcinoma (LUAD), kidney chromophobe (KICH), lung squamous cell carcinoma (LUSC), uterine corpus endometrial carcinoma (UCEC), and breast invasive carcinoma (BRCA) (Figure 1A). Paired-sample analysis corroborated high expression in CHOL, COAD, KIRC, and LIHC, but low expression in BLCA, BRCA, KICH, LUAD, LUSC, and UCEC (Figure 1B). In the TCGA-KIRC dataset, EEF1A1 mRNA levels in tumor tissues were significantly higher than in normal tissues; however, in the GSE66272, GSE36895, and GSE53757 validation cohorts, its expression was significantly lower in cancerous tissues compared to normal controls. Such discrepancies may stem from technical differences between RNA-seq and microarray platforms as well as inter-cohort heterogeneity in patient clinicopathological features and tumor purity (Figure 1C–F).

### 3.2. Correlation with Clinical Features and Prognostic Value of EEF1A1 in KIRC

**Figure 2:**
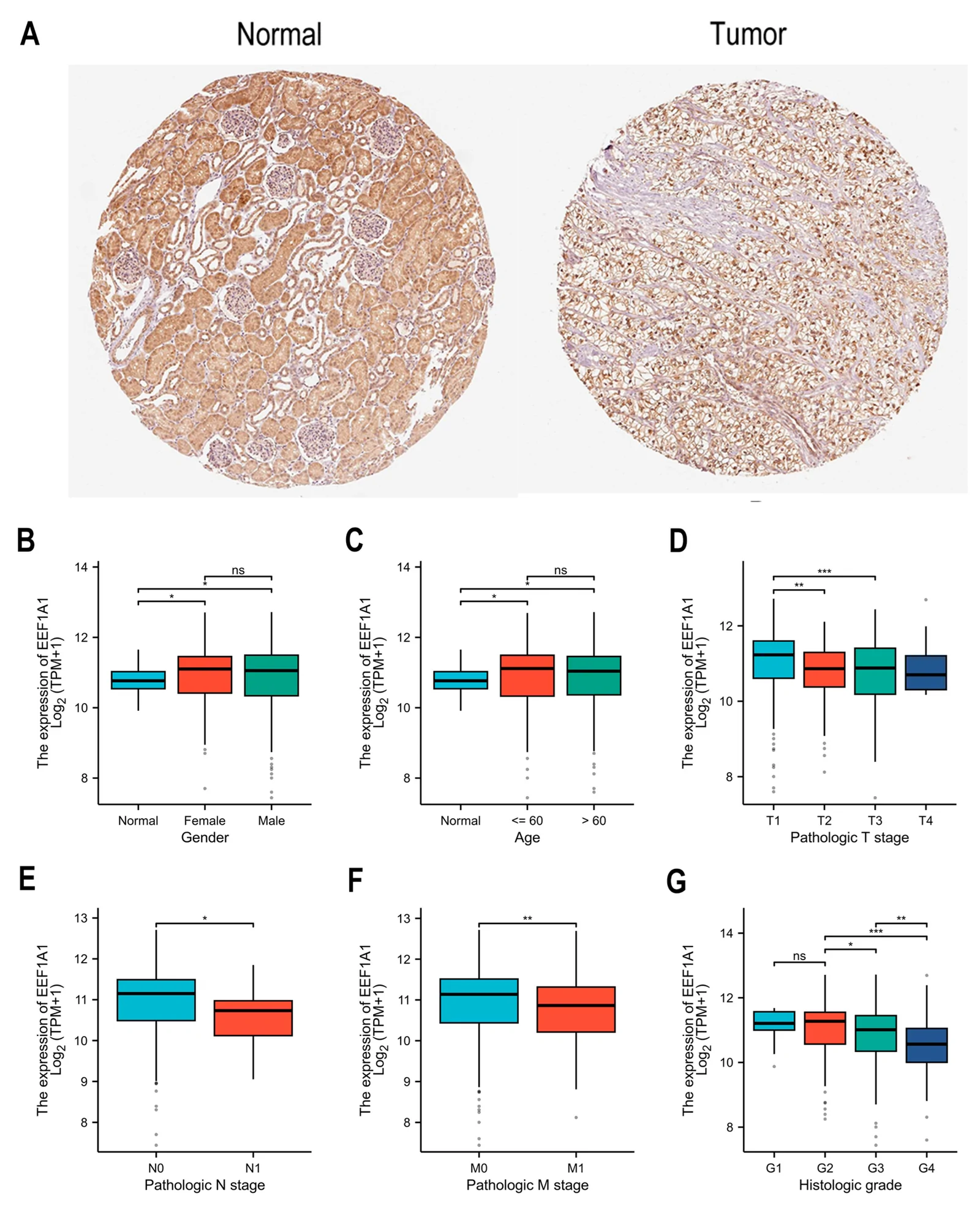
Expression of EEF1A1 in KIRC and correlation with clinical variables. (**A**) EEF1A1 protein expression levels in KIRC and paracancerous tissues in the HPA database; (**B**–**G**) graphical representation of the association between EEF1A1 mRNA expression and various clinical variables in KIRC patients, including gender (**B**), age (**C**), T stages (**D**), N stages (**E**), M stages (**F**), and pathological grades (**G**). * *p* < 0.05, ** *p* < 0.01, *** *p* < 0.001, ns, no significant difference.

**Figure 3:**
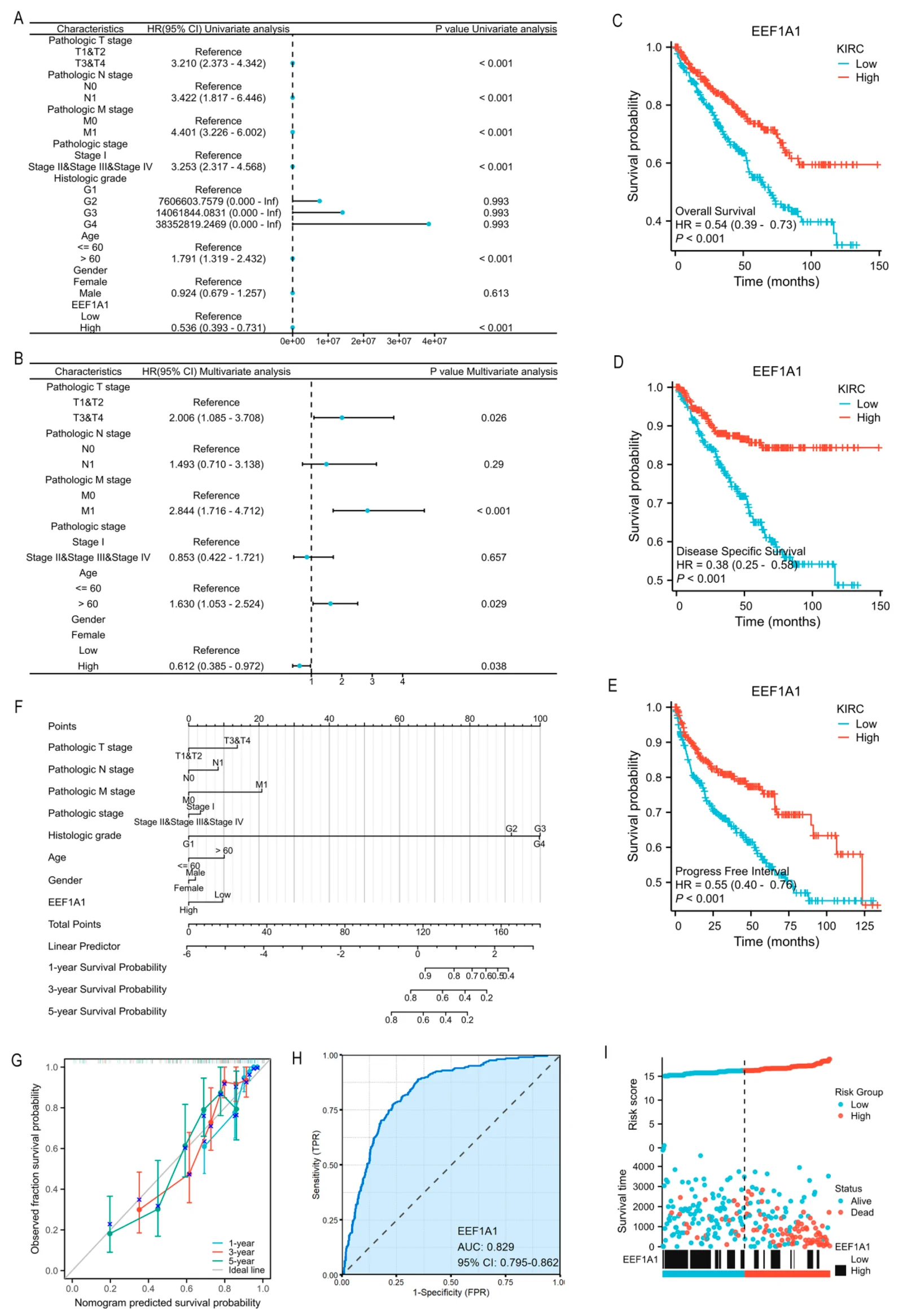
Prognostic value of EEF1A1 expression in KIRC. (**A**) Univariate Cox regression analysis of clinical pathological characteristics in KIRC patients; (**B**) multivariate Cox regression analysis of clinical pathological characteristics in KIRC patients; (**C**–**E**) Kaplan–Meier survival curves comparing the overall survival (OS), disease-specific survival (DSS), and progression-free interval (PFI) for patients with different levels of EEF1A1 expression; (**F**) prognostic nomogram based on clinical variables and EEF1A1 expression to predict overall survival; (**G**) calibration curves validating the nomogram’s predictive accuracy for KIRC patient survival of 1, 3, and 5 years; (**H**) ROC curve representing the diagnostic potential of EEF1A1 in KIRC; (**I**) risk factor plot displaying the prognostic model’s risk score and stratification.

IHC staining from the HPA database showed that EEF1A1 protein expression was weaker in KIRC tissues than in normal tissues, with localization primarily in the cytoplasm and cell membrane (Figure 2A). Correlation analysis revealed that EEF1A1 expression level was not significantly associated with patient age or gender. EEF1A1 expression exhibited a progressive downward trend as pathological T, N and M stages advanced. Specifically, EEF1A1 expression was significantly lower in T2 and T3 tumors than in T1 tumors, whereas no statistically significant differences were observed among advanced T-stage subgroups (T3 vs. T4, T2 vs. T3, T2 vs. T4, T1 vs. T4). In addition, EEF1A1 expression was markedly lower in N1 tumors compared with N0 tumors, and significantly reduced in M1 tumors relative to M0 tumors (Figure 2B–G). Univariate Cox regression analysis identified T3-T4, N1, M1, pathological grades II-IV, age, and low EEF1A1 expression as risk factors for poor prognosis in KIRC patients (Figure 3A). Multivariate Cox regression further confirmed that low EEF1A1 expression is an independent risk factor for poor prognosis (Figure 3B). Kaplan–Meier analysis demonstrated that the low-expression group had significantly worse OS, DSS, and PFI than the high-expression group (Figure 3C–E). The constructed nomogram showed high consistency between predicted and observed 1-, 3-, and 5-year survival rates (Figure 3F,G). ROC analysis indicated high diagnostic value (AUC = 0.829, Figure 3H); risk score plots suggested that lower EEF1A1 expression correlates with a higher risk of disease progression and metastasis (Figure 3I).

### 3.3. Association Between EEF1A1 Methylation and Prognosis

**Figure 4:**
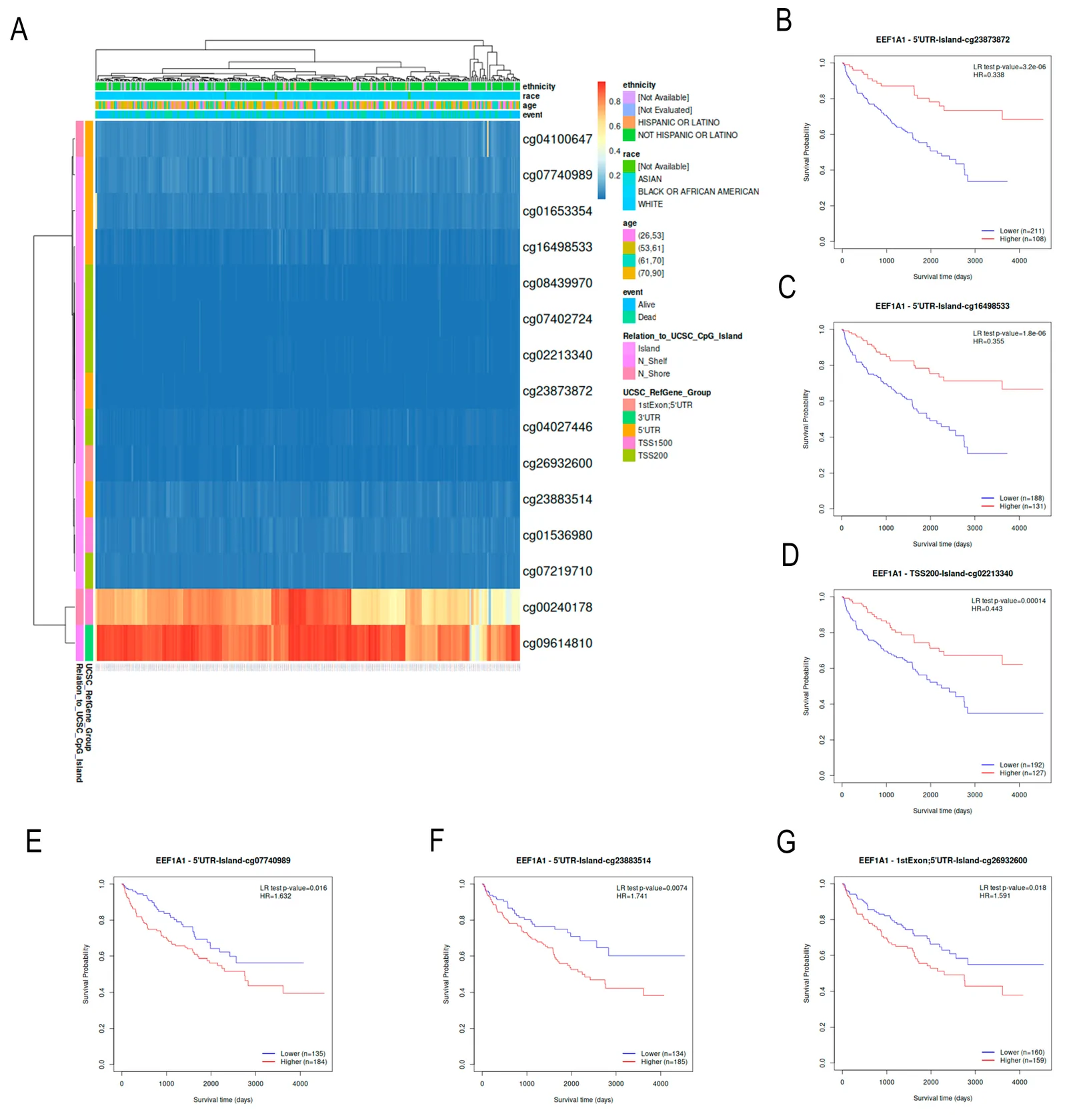
The association between EEF1A1 DNA methylation and prognosis of KIRC patients. (**A**) The association between EEF1A1 expression and methylation levels in KIRC from the MethSurv database; (**B**–**G**) Kaplan–Meier survival curves showing the prognostic value of methylation levels at the EEF1A1 CpG sites in KIRC patients.

MethSurv analysis identified 15 CpG methylation sites within the EEF1A1 gene (Figure 4A). Among these, 10 CpG sites (including cg16498533, cg23873872 and cg02213340) were associated with poor prognosis, where hypermethylation correlated with reduced survival. Conversely, 3 CpG sites (cg07740989, cg26932600, and cg23883514) were associated with favorable outcomes, where hypermethylation correlated with improved prognosis (Figure 4B–G).

### 3.4. Screening of EEF1A1-Related Genes and Enrichment Analysis of Immune-Related Pathways in Clear Cell Renal Cell Carcinoma

**Figure 5:**
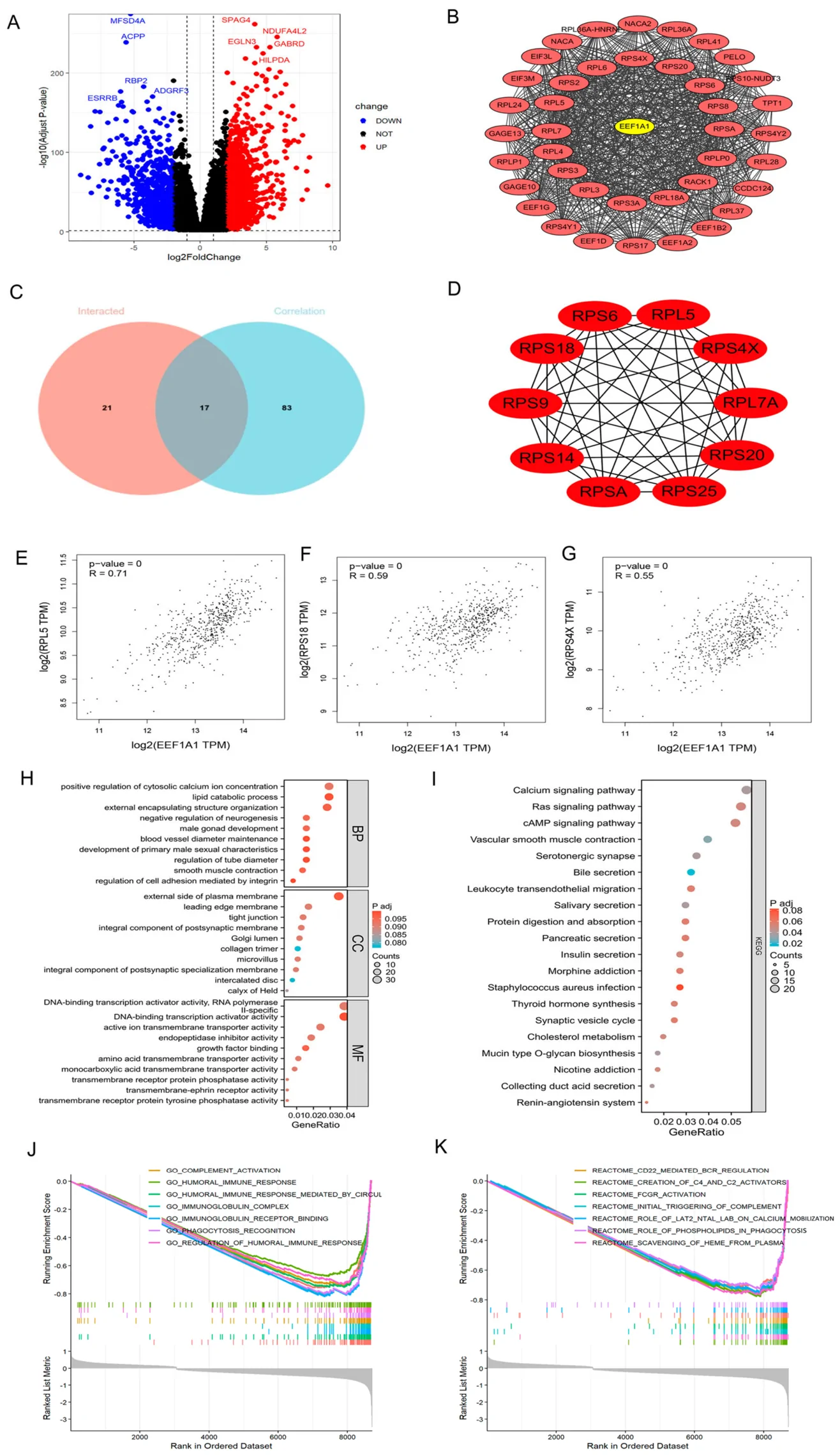
Functional enrichment analysis of EEF1A1 interaction- and expression-related genes. (**A**) Volcano plot showing the top 5 significantly up- and downregulated genes from TCGA-KIRC differential expression analysis (|log_2_FC| > 0, *p* < 0.01). (**B**) The PPI network was constructed using the STRING database with 40 proteins which were experimentally verified to bind to EEF1A1. (**C**) Venn diagram representing the intersection of EEF1A1 interaction- and expression-related genes. (**D**) Top 10 hub genes based on the MCC ranking by the CytoHubba plugin. (**E**–**G**) Expression correlation plots for the top 3 genes most strongly correlated with EEF1A1. (**H**,**I**) GO and KEGG enrichment analysis of EEF1A1 interaction and expression-related. (**J**) Gene Set Enrichment Analysis (GSEA) of TCGA-KIRC patients with low EEF1A1 expression using MSigDB C5 GO gene sets. (**K**) GSEA of the same cohort using KEGG pathway gene sets extracted from MSigDB C2. Note that pathway labels within the plot are truncated for layout constraints; their full names are “ROLE OF LAT2-NTAL IN CALCIUM MOBILIZATION” and “ROLE OF PHOSPHOLIPIDS IN PHAGOCYTOSIS”.

All analyses described in this section were performed based on the TCGA-KIRC dataset. Differential expression analysis of this profile (∣log_2_FC∣ > 0, *p* < 0.01) identified 1522 upregulated and 851 downregulated genes; the top 5 significant genes are shown in the volcano plot (Figure 5A). All differentially expressed genes (DEGs) identified in this study were imported into STRING to build the PPI network. The resulting network consisted of 41 nodes and 734 edges (Figure 5B). Intersection of the top 100 co-expressed genes from GEPIA yielded 17 overlapping genes, including RPL4, EEF1B2, and RPS4X (Figure 5C). Using CytoHubba, 10 Hub genes were extracted, with RPL5, RPS18, and RPS4X showing a strong positive correlation with EEF1A1 (Figure 5D–G). GO/KEGG analysis revealed enrichment in calcium signaling, Ras, cAMP, lipid metabolism, cell adhesion, and immune response pathways (Figure 5H,I). We conducted Gene Set Enrichment Analysis (GSEA) on MSigDB C5 Gene Ontology (GO) gene sets (Figure 5J) and KEGG pathway gene sets extracted from MSigDB C2 (Figure 5K). Notably, gene signatures involved in B-cell activation, complement activation, and antigen–antibody binding were significantly enriched in the low-EEF1A1 subgroup. Collectively, these findings imply that EEF1A1 participates in reshaping the tumor immune microenvironment (TIME).

### 3.5. Correlation of EEF1A1 with Immune Infiltration and Immunomodulators

**Figure 6:**
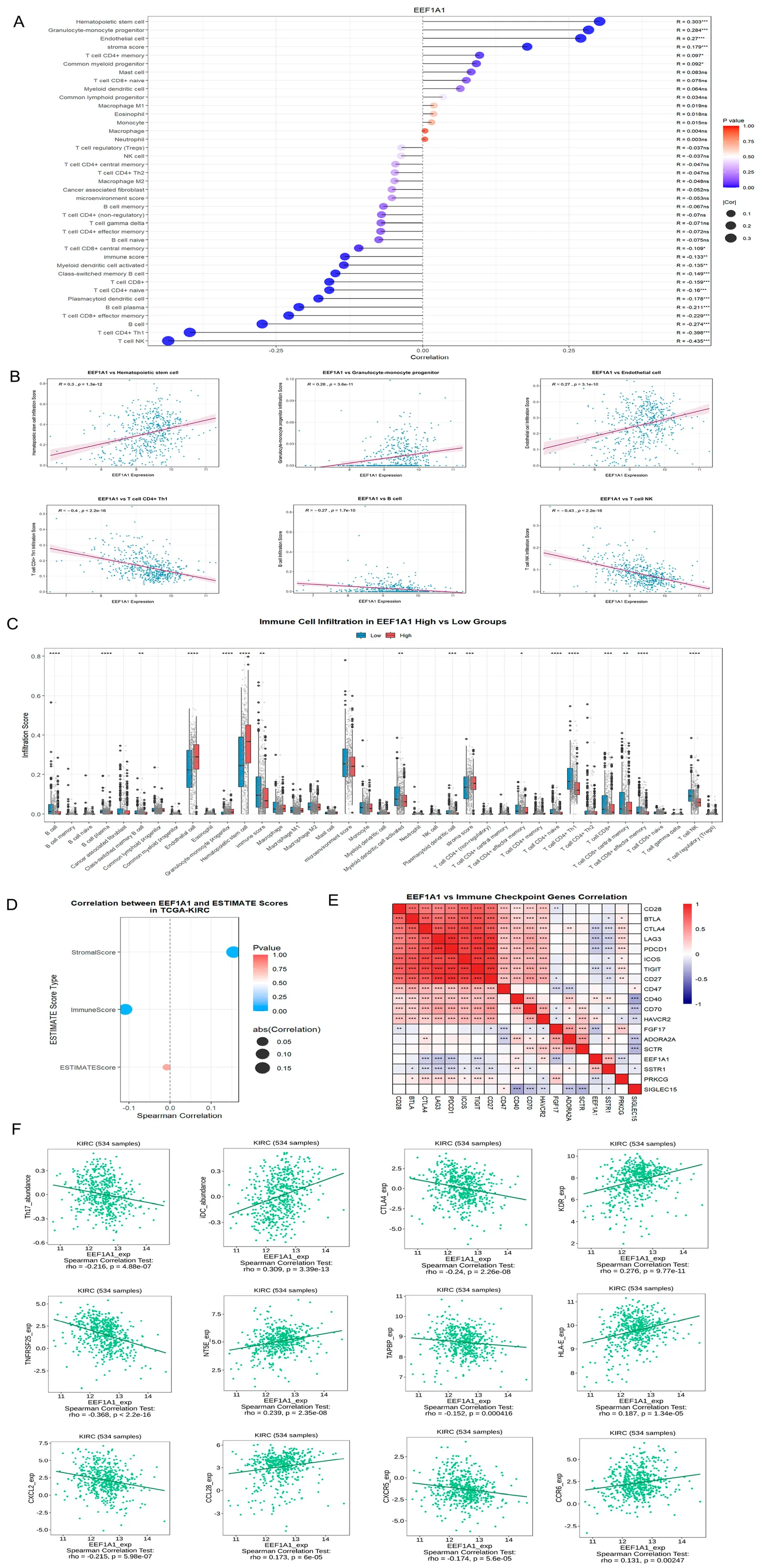
Correlation of EEF1A1 expression with immune cell infiltration and immune checkpoint in KIRC. (**A**) Lollipop plot of the correlation between EEF1A1 expression and infiltration of 33 immune cell types; (**B**) scatter plots showing the correlation between EEF1A1 expression and various immune cells; (**C**) dot plots of immune infiltration scores for high and low expression of EEF1A1; (**D**) bubble plot of the correlation between the EEF1A1 and ImmuneScore, StromalScore, and ESTIMATEScore; (**E**) heatmap of the correlation between EEF1A1 and 18 common immune checkpoint-related genes; (**F**) correlation of EEF1A1 expression with tumor-infiltrating lymphocytes (TILs), immune modulators (including immunoinhibitors immunostimulators, and MHC class-related molecules), and chemokines and their receptors. * *p* < 0.05, ** *p* < 0.01, *** *p* < 0.001, **** *p* < 0.0001, ns, no significant difference.

Using the xCell algorithm, we found that EEF1A1 expression positively correlated with 5 immune cell types (including hematopoietic stem cells and endothelial cells) and negatively with 11 others (including NK T cells, Th1, and B cells) (Figure 6A). These correlations were further quantified by scatter plots (Figure 6B). Further analysis showed that the high-expression group had significantly higher infiltration of 15 immune cell types, including monocytes, M1/M2 macrophages, and dendritic cells (Figure 6C). ESTIMATE scores positively correlated with EEF1A1 expression (Figure 6D). Immune checkpoint analysis showed significant negative correlations with CTLA4, LAG3, PDCD1, and TIGIT, but positive correlations with CD40 and HAVCR2 (Figure 6E). Data from TISIDB showed selective correlation with TILs: negative with Th17 and MDSCs, and positive with IDC and Tcm CD8. For immunomodulators, EEF1A1 negatively correlated with CTLA4 and PDCD1 but positively with KDR and IDO1. MHC analysis showed negative correlations with TAPBP and HLA-F but positive with HLA-E. Chemokine analysis revealed negative correlations with CCL2 and CCR5 but positive with CCL28 and CX3CL1 (Figure 6F).

### 3.6. Correlation with Drug Sensitivity

**Figure 7:**
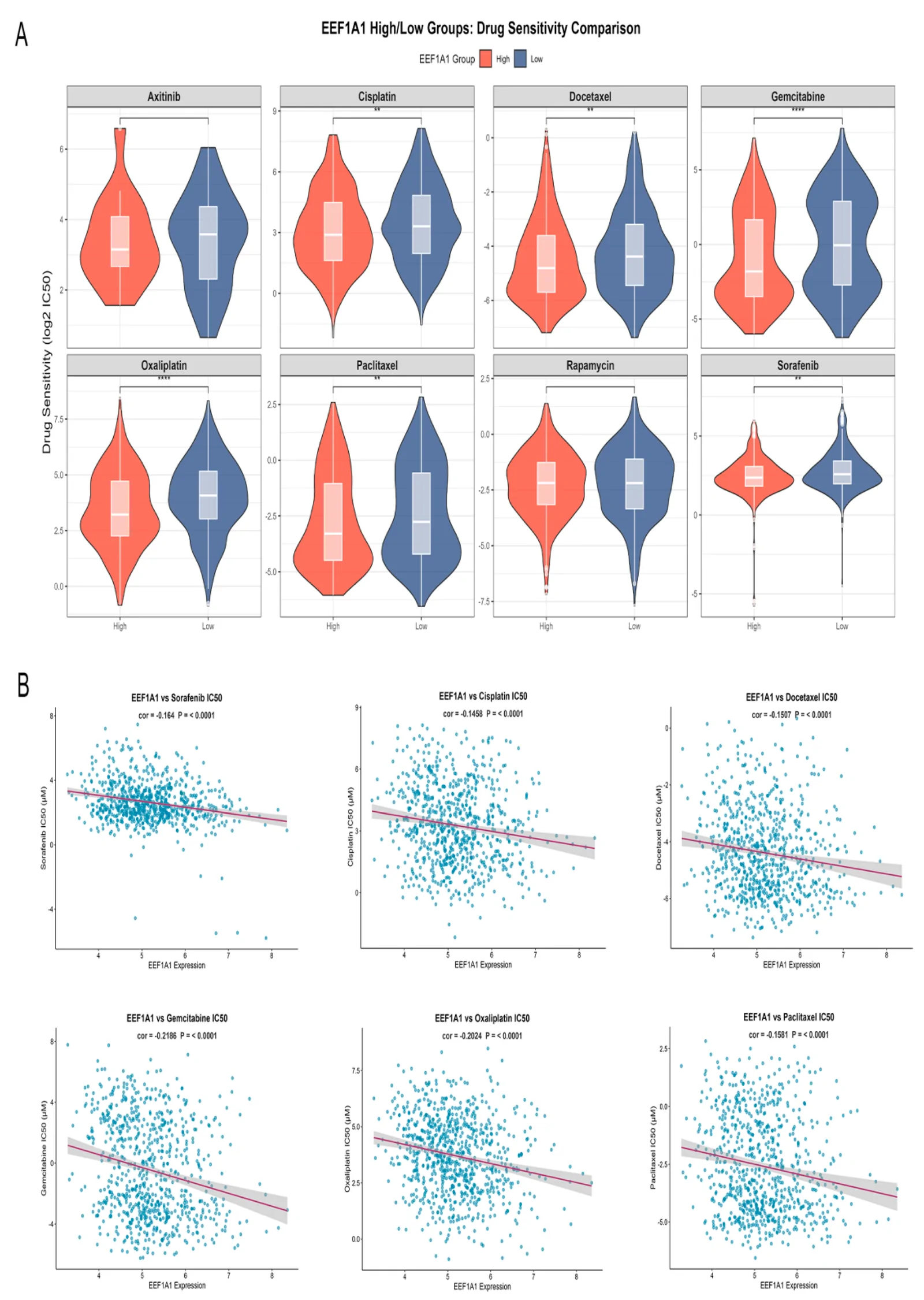
Drug sensitivity analyses. (**A**) Violin plots combined with boxplots show the distribution of log2-transformed IC50 values for 8 commonly used anticancer drugs (axitinib, cisplatin, docetaxel, gemcitabine, oxaliplatin, paclitaxel, rapamycin, and sorafenib) in the EEF1A1 high-expression (red) and low-expression (blue) groups. A higher log2 IC50 value indicates lower drug sensitivity (greater drug resistance), while a lower value indicates higher sensitivity. (**B**) The relationships between EEF1A1 expression levels and the IC50 values (μM) of six anticancer drugs, including sorafenib, cisplatin, docetaxel, gemcitabine, oxaliplatin, and paclitaxel. Statistical significance between the two groups was assessed, and significance levels are marked as ** *p* < 0.01, **** *p* < 0.0001. Red line represents the linear regression fit; grey shaded area indicates the 95% confidence interval.

CellMiner™ analysis showed that the high EEF1A1 expression group was significantly more sensitive to six common drugs, including sorafenib, paclitaxel, and cisplatin (Figure 7A). Scatter plots with linear regression lines confirmed a significant negative correlation between EEF1A1 expression and the IC50 values of these drugs (Figure 7B).

### 3.7. Verification of EEF1A1 Expression and Its Impact on Biological Behavior of KIRC

**Figure 8:**
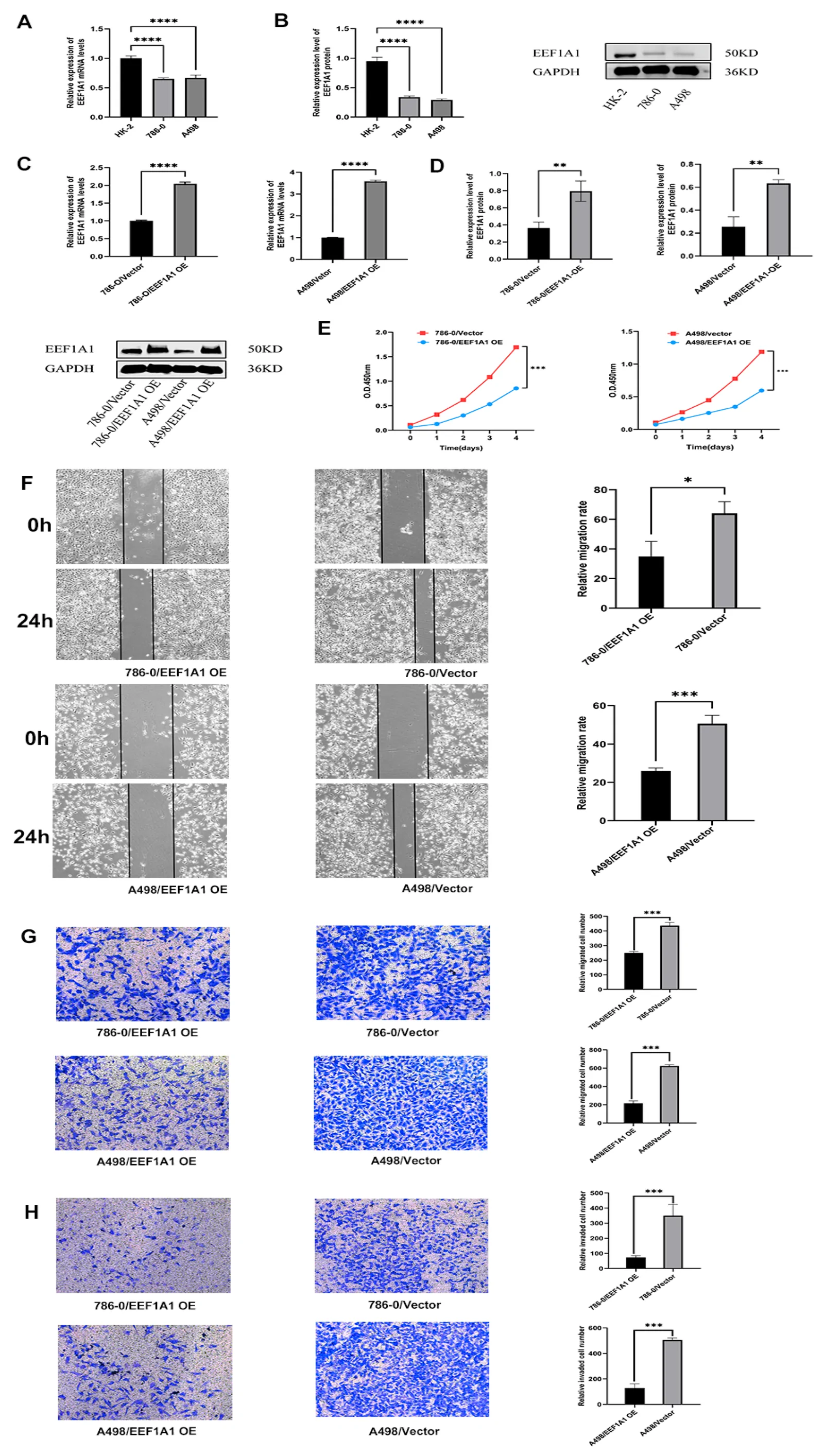
Validation of EEF1A1 expression and its impact on the proliferation, migration, and invasion of kidney renal clear cell carcinoma. (**A**,**B**) Expression levels of EEF1A1 mRNA and protein in human renal tubular epithelial cells (HK-2) and renal clear cell carcinoma cell lines (786-0 and A498) (Supplementary Materials S1). (**C**,**D**) The overexpression efficiency of EEF1A1 in renal clear cell carcinoma cell lines (786-0 and A498) was assessed using quantitative real-time polymerase chain reaction (qRT-PCR) and Western blotting (Supplementary Materials S2). (**E**) CCK-8 assay was used to evaluate cell proliferation. (**F**) Wound healing assay was performed to determine cell migration capability. (**G**,**H**) Transwell migration and invasion assays were used to evaluate the migratory and invasive capabilities of cells, respectively. Data are presented as the mean ± SD, with n = 3 independent biological replicates for each experiment. Statistical significance between the two groups was assessed, and significance levels are marked as * *p* < 0.05, ** *p* < 0.01, *** *p* < 0.001, **** *p* < 0.0001.

The qRT-PCR results showed that EEF1A1 mRNA levels were significantly upregulated in 786-0 and A498 cells compared to HK-2 cells (Figure 8A). Western blotting revealed that the protein levels of EEF1A1 were significantly lower in cancer cells than in HK-2 cells (Figure 8B). Following the construction of EEF1A1-stably overexpressing 786-0 and A498 cells, qRT-PCR and Western blot assays confirmed the overexpression efficiency (Figure 8C,D). CCK-8 assays indicated that the proliferative ability of renal clear cell carcinoma cells was significantly decreased after EEF1A1 overexpression (*p* < 0.001) (Figure 8E). Wound healing assays demonstrated a significant reduction in the migratory ability of renal clear cell carcinoma cells (*p* < 0.001) (Figure 8F). Transwell migration and invasion assays further corroborated the role of EEF1A1 in cell motility, showing that the number of migrated and invaded cells was substantially reduced upon EEF1A1 overexpression (*p* < 0.001) (Figure 8G,H,). These results confirm the critical role of EEF1A1 in the proliferation, migration, and invasion of renal clear cell carcinoma cells.

## 4. Discussion

Eukaryotic translation elongation factor 1 alpha 1 (EEF1A1) is a core member of the eukaryotic translation elongation factor family. Its canonical role involves binding aminoacyl-tRNA complexes to facilitate peptide chain elongation on ribosomes during protein synthesis [3,15]. EEF1A1 is expressed in various human tissues, including the brain, lung, liver, kidney, and pancreas, and its aberrant expression plays a critical role in the tumorigenesis and progression of multiple cancers [16,17,18]. However, the specific role and underlying molecular mechanism of EEF1A1 in KIRC remain unclear. In this study, we systematically analyzed the expression profile, clinical significance, and potential molecular mechanism of EEF1A1 in KIRC based on public databases including TCGA and GEO. The results demonstrated that EEF1A1 was significantly downregulated in KIRC tissues and was closely associated with tumor TNM stage, pathological grade, and overall survival. High expression of EEF1A1 was identified as an independent favorable prognostic factor for KIRC patients. Functional enrichment analysis suggested that EEF1A1 may participate in tumor progression by regulating metabolic reprogramming, Ras/cAMP signaling, cytoskeleton regulation, and immune-related pathways. In the tumor microenvironment (TME) of KIRC, EEF1A1 was significantly correlated with multiple immune cell infiltration, immune checkpoints, tumor-infiltrating lymphocytes, and various immunomodulatory genes. Furthermore, high expression of EEF1A1 could markedly enhance the sensitivity of KIRC cells to clinically common drugs such as sorafenib, and its expression level was negatively correlated with drug inhibitory activity. Collectively, EEF1A1 is expected to serve as a potential biomarker for improving prognosis and guiding precise immunotherapy in KIRC patients.

In this study, we identified 10 hub genes interacting with EEF1A1, including RPL5, RPS4X, and RPS14. Most of these genes encode ribosomal proteins and exert oncogenic or tumor-suppressive roles in various cancers, such as breast cancer, glioblastoma, bladder cancer, and myelodysplastic syndromes, by regulating the p53 and c-MYC pathways or DNA repair mechanisms [18,19,20,21]. GO and KEGG enrichment analyses revealed that EEF1A1 and its co-expressed genes were mainly involved in metabolic regulation (such as the switch between glycolysis and oxidative phosphorylation), maintenance of ion homeostasis, cytoskeleton remodeling (e.g., regulation of E-cadherin), and the Ras/cAMP signaling pathway [22,23,24,25,26]. Furthermore, in terms of immune regulation, EEF1A1 can directly bind to 2’3’-cGAMP to activate the STING (Stimulator of Interferon Genes) pathway, which subsequently recruits dendritic cells and CD8+ T cells to initiate innate anti-tumor immunity [27]. It is therefore speculated that EEF1A1 may affect the tumorigenesis and progression of KIRC from multiple dimensions, including metabolism, cellular structure, and signal transduction, by constructing a complex regulatory network. GSEA revealed significant enrichment of dysregulated immune-related pathways in the EEF1A1-low subgroup, including perturbed B-cell activation, an altered complement system, and disrupted cytoskeletal reorganization. Complement and B-cell-mediated humoral immune responses exert dual effects in ccRCC: moderate complement activation promotes anti-tumor immunity, while sustained complement signaling drives immunosuppressive remodeling of the tumor microenvironment. Combined with our prior finding that low EEF1A1 expression correlates with poor clinical prognosis, these enrichment results suggest that EEF1A1 downregulation may trigger aberrant humoral immune activation within ccRCC lesions. Furthermore, ribosomal hub genes (RPL5, RPS18, and RPS4X) identified from the PPI network are positively correlated with EEF1A1. Given the known crosstalk between ribosomal proteins and tumor immune signaling, we hypothesize that EEF1A1 may cooperate with these hub molecules to remodel the TIME. Collectively, these bioinformatic observations support the oncogenic role of EEF1A1 in ccRCC, which is further validated by our in vitro functional assays.

Previous studies have confirmed that EEF1A1 participates in regulating antigen presentation, translation efficiency, and hypoxia-induced immunosuppression, thereby promoting CD8^+^T cell-mediated tumor killing [28,29,30]. Inhibition of EEF1A1 can reduce PD-L1 expression, reverse the immunosuppressive tumor microenvironment, restore immune surveillance, and activate anti-tumor immunity [26]. Our immune infiltration analyses revealed significant associations between EEF1A1 expression and multiple immune cell populations in KIRC. These findings suggest that EEF1A1 may be involved in immune-related processes within the tumor microenvironment; however, further experimental studies are required to determine whether EEF1A1 directly regulates immune cell recruitment or function. Correlation analysis between EEF1A1 and the tumor immune microenvironment (TIME) of KIRC revealed that EEF1A1 expression was positively correlated with hematopoietic stem cells, CD4^+^ memory T cells, and other immune cell subsets. Moreover, the infiltration levels of M1-type macrophages, NK cells, and dendritic cells were significantly elevated in the EEF1A1 high-expression group. These findings suggest that EEF1A1 may participate in remodeling the tumor immune microenvironment, enhancing local immune surveillance, regulating the recruitment and distribution of lymphocytes, myeloid cells and stromal-derived immune cells, as well as curbing immune evasion and the establishment of an immunosuppressive state, thereby exerting tumor-suppressive effects in KIRC [31,32,33,34,35,36]. Regarding immune checkpoints, EEF1A1 was significantly negatively correlated with multiple key inhibitory immune checkpoints, including CTLA4, LAG3, PDCD1, and TIGIT, but positively correlated with the co-stimulatory molecule CD40. High expression of EEF1A1 may reduce T-cell exhaustion by downregulating these key inhibitory immune checkpoints and restore the anti-tumor immune functions of effector T cells, NK cells, and other immune cells, thereby inhibiting tumor progression [37,38,39,40,41,42]. CD40 contributes to antigen presentation, activation of T and B cells, and enhancement of immune memory and immune surveillance, and its high expression is closely associated with strengthened anti-tumor immunity [43,44]. In terms of tumor-infiltrating lymphocytes (TILs) and immunoregulatory factors, high EEF1A1 expression modulated the infiltration of tumor immune cells, including MDSCs, IDCs, Tgd cells, and CD4^+^Tem cells, enhanced CD8^+^T-cell function, and suppressed the formation of an immunosuppressive microenvironment [7,28,45,46]. Furthermore, EEF1A1 regulated the expression of MHC molecules (HLA-F, HLA-DOB, HLA-E, and HLA-DRA), chemokines (CCL2, CCL5, CCL28, CX3CL1, etc.) and their receptors (CCR5, CCR8, CCR6, CX3CR1, etc.), inhibited immunosuppressive signal transduction, strengthened anti-tumor immune responses, and reshaped the landscape of tumor immune cell infiltration. In this way, EEF1A1 constructs an anti-tumor immune microenvironment characterized by low suppression, high surveillance, and stable homeostasis, thus playing a tumor-suppressive role in KIRC [47,48,49,50,51,52,53]. These findings also provide a solid theoretical basis for EEF1A1 as a potential target for immunotherapy of KIRC.

Another significant highlight of this study is the exploration of EEF1A1 as a potential clinical biomarker for predicting precision drug sensitivity in KIRC. Specifically, high EEF1A1 expression significantly enhanced the sensitivity of KIRC cells to agents targeting the cell cycle (e.g., paclitaxel) and DNA damage repair pathways, while showing no significant association with mTOR inhibitors or immunotherapy. These findings suggest that EEF1A1 may differentially regulate apoptotic pathways or DNA damage checkpoints through its canonical translation elongation function. This discovery not only provides a novel predictive indicator for individualized chemotherapy in KIRC patients but also offers a new breakthrough for overcoming drug resistance and identifying sensitization targets in advanced renal cancer.

To further elucidate the functional role of EEF1A1, we conducted in vitro functional assays using renal clear cell carcinoma (786-0 and A498) cell lines. Our results demonstrated that EEF1A1 overexpression significantly inhibited the proliferation, migration, and invasion capabilities of renal clear cell carcinoma cells. These findings provide a solid experimental basis for revealing the specific mechanism of EEF1A1 in the development and progression of kidney renal clear cell carcinoma, while also offering important theoretical references and experimental evidence for future research.

However, we acknowledge certain limitations in this study. First, in vivo animal experiments are currently lacking to further validate the impact of EEF1A1 on the biological behavior of KIRC tumors. Second, although we explored the function and value of EEF1A1 in immune infiltration through bioinformatics analysis, we have not yet experimentally validated the related metabolic and immune pathways it regulates, nor the specific interaction mechanisms with immune cells. Finally, the upstream and downstream regulatory relationships between EEF1A1 and specific signaling pathways have not been fully explored in this study, and the relevant molecular mechanisms remain to be further elucidated by future functional experiments.

## 5. Conclusions

In summary, EEF1A1 is significantly downregulated in KIRC tissues and contributes to tumor progression. Its expression level is closely associated with TNM stage, pathological grade and clinical prognosis of KIRC patients. Mechanistically, the interacting proteins and co-expressed genes of EEF1A1 are implicated in multiple metabolic and immune pathways and correlate with the infiltration of various immune cells. Collectively, EEF1A1 represents a promising biomarker for evaluating prognosis, immune microenvironment status and drug sensitivity in KIRC, and provides a novel therapeutic target for overcoming drug resistance in advanced KIRC.

## Figures and Tables

**Table 1:** The sequence of primers.

Gene	Forward Primer (5′–3′)	Reverse Primer (5′–3′)
*EEF1A1*	ACCACTACTGGCCATCTGATCTA	GTTTATCCAAGACCCAGGCATAC
*GAPDH*	AAGCTCATTTCCTGGTATGACAA	CTTACTCCTTGGAGGCCATGT

## Data Availability

The datasets used and/or analyzed during the current study are available from the corresponding author on reasonable request.

## Supplementary Materials

The following supporting information can be downloaded at: https://www.mdpi.com/article/10.3390/cancers18182938/s1.
